# Molecular Profiles and Antimicrobial Resistance Genes in Bacterial Isolates from Chronic Rhinosinusitis Patients

**DOI:** 10.3390/pathogens15030311

**Published:** 2026-03-12

**Authors:** Andrei Osman, Alice Elena Ghenea, Carmen Aurelia Mogoanta, Irina Enache, Alexandra Bucătaru, Ramona Cioboată, Mădălina Georgescu, Andrei Theodor Bălășoiu, Ovidiu Mircea Zlatian

**Affiliations:** 1 Department of Anatomy and Embriology, University of Medicine and Pharmacy of Craiova, 200349 Craiova, Romania; andrei.osman@umfcv.ro (A.O.); irina.enache@umfcv.ro (I.E.); 2 Department of Microbiology, University of Medicine and Pharmacy of Craiova, 200349 Craiova, Romania; alice.ghenea@umfcv.ro (A.E.G.); ovidiu.zlatian@umfcv.ro (O.M.Z.); 3 Department of Otorhinolaryngology, University of Medicine and Pharmacy of Craiova, 200349 Craiova, Romania; carmen.mogoanta@umfcv.ro; 4 Department of Pneumology, University of Medicine and Pharmacy of Craiova, 200349 Craiova, Romania; ramona_cioboata@yahoo.rcom; 5 Department of General Surgery and Qualified Care in Surgical Specialties, “Carol Davila” University of Medicine and Pharmacy, 050474 Bucharest, Romania; 6 Department of Ophthalmology, University of Medicine and Pharmacy of Craiova, 200349 Craiova, Romania; andrei_theo@yahoo.com

**Keywords:** chronic rhinosinusitis, bacterial pathogens, antimicrobial resistance, antibiotic resistance, resistance genes, *Staphylococcus aureus*, *Klebsiella pneumoniae*, *Pseudomonas aeruginosa*, *tem*, *temB*, *ctxM*, *shv*, *sul1*, *ermB*, *mecA*

## Abstract

(1) Background: Chronic rhinosinusitis (CRS) with recurrent symptoms despite therapy raises concern for underlying antimicrobial resistance. While inflammation is central to disease pathophysiology, increasing evidence suggests that resistant bacterial populations within the sinonasal niche may contribute to treatment failure. This study aimed to characterize the molecular resistance profiles of bacterial isolates from refractory CRS patients and evaluate genotype–phenotype concordance and clinical resistance burden. (2) Methods: Our observational study includes 99 bacterial isolates obtained by endoscopically guided nasal swabs from adult CRS patients with recurrent disease. Species identification and antimicrobial susceptibility testing were performed using the VITEK^®^2 system. Resistance genes were detected using multiplex-PCR. Statistical analyses included Mann–Whitney U tests for genotype–phenotype associations, Kruskal–Wallis testing across MDR categories, Spearman correlation between gene burden and clinical risk, and concordance metrics. (3) Results: Recognized sinonasal pathogens accounted for 46.5% of isolates, predominantly *Staphylococcus aureus*, *Klebsiella pneumoniae*, *Proteus mirabilis*, and *Pseudomonas aeruginosa*. β-lactamase genes (*tem* 25.3%, *shv* 9.1%, *ctxM* 8.1%) and macrolide resistance markers (*ermB* 20.2%) were most prevalent, while carbapenemase genes remained infrequent. Significant phenotype–genotype correlations were observed for *mecA*–oxacillin, *sul1*–TMP-SMX, *KPC*–meropenem, and *tem*–β-lactams (*p* < 0.01). Gene burden increased progressively across clinical risk categories (*p* < 0.001), with MDR/XDR isolates concentrated in patients with repeated antibiotic exposure. Molecular and phenotypic analyses demonstrated high concordance for selected gene–antibiotic pairs, supporting targeted molecular screening as an adjunct to culture-based diagnostics in refractory CRS.

## 1. Introduction

CRS significantly impacts the quality of life (QoL) of affected individuals, manifesting through both physical and psychological symptoms [1]. The condition is prevalent worldwide, affecting a substantial portion of the population [2], and is characterized by persistent inflammation of the sinonasal mucosa [2]. The frequency and severity of CRS symptoms, such as nasal obstruction, facial pain, nasal discharge and reduced sense of smell [3], contribute to a diminished QoL [4,5], comparable to other chronic life-long diseases like asthma or angina [6,7].

The pathophysiology of CRS has evolved significantly, with recent advancements providing a deeper understanding of its complex mechanisms. CRS is now recognized as a multifactorial inflammatory condition, characterized by a spectrum of immune responses and influenced by genetic, environmental, and microbial factors. CRS is increasingly understood as a disease spectrum with distinct inflammatory endotypes, primarily categorized into type 2 (eosinophilic) and non-type 2 inflammatory responses [8,9,10]. The role of eosinophils and microbial pathogens such as *Staphylococcus aureus* in the persistence of CRS has been explored, with the given bacteria acting as a disease modifier in certain patient subsets [11,12,13].

The development of CRS following acute sinusitis (AS) is a complex process influenced by various factors, where the microbiological pathogens might play a key role [14]. AS often begins with a viral infection, which can lead to bacterial colonization and infection of the nasal mucosa and paranasal sinuses. The transition to CRS involves persistent inflammation and the establishment of a polymicrobial environment, including both aerobic and anaerobic bacteria [15,16]. This transition is further complicated by factors such as biofilm formation, development of antibiotic resistance and host immune responses [17,18].

The use of antibiotics in treating AS can lead to the selection of resistant bacterial strains, which are more prevalent in CRS patients [19]. This resistance is often due to the presence of beta-lactamase-producing bacteria and the protective environment of biofilms [17,20,21]. In addition to biofilm formation, bacterial genetics may play an important role in the persistence of certain strains in the sinonasal environment [22]. Bacterial resistance genes like tem, which confers broad-spectrum penicillin resistance, are often identified in pathogens causing upper respiratory disease [23].

Methicillin-resistant *Staphylococcus aureus* is perhaps one of the best-known pathogens in CRS due to its resistance to methicillin and other beta-lactam antibiotics. This resistance is primarily mediated by the *mecA* gene, which alters the target site of beta-lactam antibiotics, rendering them ineffective [24]. *Pseudomonas aeruginosa*, particularly in anaerobic conditions, shows increased resistance to antibiotics such as gentamicin and levofloxacin. This resistance is also linked to biofilm formation, which protects the bacteria from the effects of antibiotics [25]. Recent studies show that the unwarranted use of antibiotics contribute to bacterial pathogens actively developing resistance genes [26]. Despite promising leads into bacterial genetics, few studies carry out molecular profiling of pathogenic bacteria, and fewer attempt correlations between bacterial populations identified in CRS patients and their total gene burden [27,28].

## 2. Materials and Methods

This observational study was conducted on patients diagnosed with CRS who experienced recurrent or severe exacerbations despite guideline-based medical management. The present study took place between September 2024 and November 2024 at the Emergency County Hospital of Craiova, in southwestern Romania. Patients were recruited either during outpatient consultations at the Otorhinolaryngology Department of the Clinical Emergency County Hospital in Craiova, or when admitted for therapy and/or endoscopic sinus surgery. Informed consent for study participation was obtained from each individual patient, archived and attached to their patient file and from the Ethics Committee of our hospital—Institutional Review Board of the Emergency Clinical County Hospital of Craiova, Romania (no. 2870/22 July 2024). The funding for this research was provided by The University of Medicine and Pharmacy in Craiova, Romania, grant number 298/701/17/01.07.2024.

Eligible patients were adults between 31 and 51 years old, with a history of CRS diagnosed according to the European Position Paper on Rhinosinusitis and Nasal Polyps 2020 criteria [29], who presented with persistent or relapsing symptoms. Typical symptoms included facial pressure, persistent nasal discharge, nasal obstruction, fetid secretions, and a history of antibiotic therapy for previous episodes of acute rhinosinusitis. Patients with a history of maxillary sinus punctures were also admitted. Exclusion criteria included:

- Recent antibiotic treatment, intranasal saline irrigation, or topical corticosteroid use within the past 30 days;
- Immunocompromising conditions (diabetes mellitus, HIV infection, long-term systemic corticosteroid therapy, malignancy, chemotherapy);
- Sinus surgery within the preceding 6 months.

Nasal swab specimens were collected in the morning, prior to any local or systemic therapy. Sampling was performed under endoscopic guidance (Karl Storz GmbH, Tuttlingen, Germany) to ensure precise targeting of the middle meatus or diseased sinus mucosa. Sterile transport swabs were immediately transferred to the microbiology laboratory, with a maximum transport interval of under two hours between collection and processing, to minimize contamination and bacterial overgrowth.

To accommodate slow-growing organisms, cultures were maintained for a period of 5 to 7 days prior to being designated as negative, with daily assessment of bacterial growth under standardized incubation conditions of 37 °C, in both aerobic and microaerophilic atmospheric environments. Primary inoculation of clinical specimens was carried out onto a panel of standard and selective culture media selected to support the optimal growth of a broad spectrum of pathogenic microorganisms. The media employed Columbia blood agar, utilized as a non-selective basal medium facilitating the growth of the majority of clinically relevant bacteria and enabling the visualization of hemolytic activity, alongside chocolate agar, specifically suited for the cultivation of fastidious microorganisms. Selective media were additionally inoculated, including MacConkey agar, employed for the isolation and differential characterization of Enterobacteriaceae and non-glucose-fermenting Gram-negative bacteria.

Standardized microbial suspensions at 0.5 McFarland turbidity were used to inoculate miniaturized biochemical reagent cards, enabling confirmation of bacterial identification through automated phenotypic profiling via the VITEK 2 system (bioMérieux, Marcy-l’Étoile, France, identification cards: GN, GP, ANC, NH; antibiotic susceptibility testing cards: AST-P592 for Gram-positive organisms, and AST-N438 and AST-XN26 for Gram-negative organisms). Preliminary bacterial identification preceded this confirmatory step and was established on the basis of colonial morphological characteristics, including size, shape, pigmentation, surface texture, and hemolytic properties, complemented by microscopic examination of Gram-stained smears prepared from pure cultures and supplemented by rapid biochemical testing. Antimicrobial susceptibility testing results were interpreted according to the Clinical and Laboratory Standards Institute (CLSI, M100 standard, 2024).

The Multiplex PCR cartridge panels employed in the present study targeted a broad spectrum of clinically relevant Gram-positive and Gram-negative bacteria associated with respiratory tract infections, simultaneously reporting a comprehensive array of antimicrobial resistance (AMR) markers, encompassing methicillin resistance determinants (*mecA*/*mecC*), glycopeptide resistance genes (*vanA*/*vanB*), extended-spectrum β-lactamases (*tem*, *shv*, *ctx-M*), aminoglycoside resistance genes (*aac2*, *aacAc4*), plasmid-mediated quinolone resistance determinants (*qnrA/B/S*, *gyrA83/87*), macrolide resistance genes (*ermA*, *ermC*), sulfonamide resistance (*sul1*), and carbapenemase-encoding genes (*kpc*, *ndm*, *vim*, *oxa-23*, *oxa-24/40*, and *oxa-48*). The Unyvero system (Curetis GmbH, Holzgerlingen, Germany) achieves pathogen identification and resistance profiling through the detection of genetic target sequences associated with specific bacterial species and molecular antibiotic resistance markers, with the entire procedure completed within approximately five hours. The analytical workflow was based on high-multiplex PCR followed by array hybridization, with results interpreted using the C8 Cockpit software interface, version 6.0. Sample processing was performed on the A50 Analyzer using application-specific cartridges, following an initial lysis step carried out in the Unyvero L4 Lysator (approximately 30 min) after sample suspension in the designated Unyvero sample tube. All collected specimens were subjected to molecular analysis using the Unyvero platform (Curetis/OpGen) [30].

Resistance profiles were interpreted in accordance with Clinical and Laboratory Standards Institute (CLSI) guidelines. Bacterial resistance was defined according to the definitions derived from Magiorakos et al., 2012 (Clinical Microbiology and Infection, ESCMID/ECDC consensus) [31]:

1. Single-Drug Resistant (SDR): Non-susceptibility to at least one antimicrobial agent in a single antimicrobial category;
2. Multidrug Resistant (MDR): Non-susceptibility to at least one agent in ≥3 different antimicrobial categories;
3. Extensively Drug Resistant (XDR): Non-susceptibility to at least one agent in all but ≤2 antimicrobial categories, i.e., the isolate remains susceptible to only one or two categories;
4. Pan-Drug Resistant, rarely reported (PDR): Non-susceptibility to all agents in all antimicrobial categories.

Each isolate was categorized into one of six predefined clinical risk categories, namely low risk, moderate risk, MRSA, extended-spectrum β-lactamase (ESBL) producer, carbapenemase producer, and High-Risk MDR [15,31,32,33]. This classification was based on phenotypic resistance profiles and molecular detection of key resistance genes. The analyzed group thus provides a representative overview of the microbial and genetic spectrum encountered in refractory or treatment-resistant CRS, serving as the basis for subsequent analyses of pathogen frequency, resistance gene distribution, and multidrug resistance patterns.

All microbiological findings, resistance profiles, and patient-related variables were systematically recorded. Patient files and clinical reports were maintained using Microsoft Word 2019 (Microsoft Corporation, Redmond, WA, USA). A dedicated database was constructed in Microsoft Excel 2019 (Microsoft Corporation, Redmond, WA, USA), which included demographic data, sample source, bacterial isolates, resistance gene profiles, and multidrug resistance status. Mann–Whitney U tests (one-tailed) compared MIC values between gene-positive and gene-negative isolates for each biologically plausible gene–antibiotic pair. Kruskal–Wallis H tests assessed MIC variation across MDR categories (Susceptible, SDR, MDR, XDR). Spearman’s rank correlations evaluated the association between total resistance gene count and individual antibiotic MIC values. Fisher’s exact tests assessed the association between individual gene carriage and MDR status (MDR/XDR/PDR vs. Susceptible/SDR). Concordance was calculated using 2 × 2 contingency tables comparing molecular gene presence with phenotypic resistance, with sensitivity, specificity, PPV, and NPV derived from standard diagnostic test formulas. CLSI 2025 breakpoints were used for phenotypic resistance classification. All analyses were performed using Python 3 with SciPy v1.11 and Pandas v2.1. Statistical significance was defined as *p* < 0.05, with Bonferroni-corrected thresholds noted where applicable.

## 3. Results

**Figure 1:**
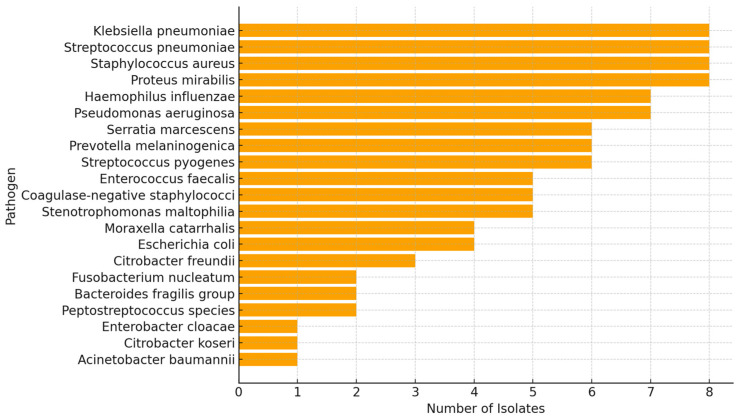
Frequency of pathogen species detected in nasal samples.

Analyzing the samples collected for this study, we identified a well-defined subset of bacterial isolates which represented a total of 99 bacterial isolates. The dataset comprises 21 distinct bacterial species, reflecting a broad microbial diversity within the sinonasal cavity of patients diagnosed with CRS. Within this cohort, a diverse bacterial community was identified, encompassing both Gram-positive and Gram-negative microorganisms (Figure 1). To better contextualize the clinical significance of these findings, isolates were stratified into recognized nasal pathogens and commensal/colonizing flora.

Pathogenic isolates.

Among species with established pathogenic potential in sinonasal infections, the most frequently identified were *Staphylococcus aureus* (n = 8, 8.1%), *Streptococcus pneumoniae* (n = 8, 8.1%), *Haemophilus influenzae* (n = 7, 7.1%), *Klebsiella pneumoniae* (n = 8, 8.1%), *Pseudomonas aeruginosa* (n = 7, 7.1%), and *Proteus mirabilis* (n = 8, 8.1%). Together, recognized pathogens accounted for 46.5% (n = 46/99) of all isolates. Notably, *Staphylococcus aureus* isolates were frequently associated with MRSA clinical categories (75% *mecA*-positive), whereas *Klebsiella pneumoniae* and *Proteus mirabilis* were predominant among samples exhibiting ESBL phenotypes (62.5% and 50.0%, respectively).

Commensal and colonizing flora.

The remaining 47.5% (n = 47/99) of isolates comprised species typically regarded as nasal commensals or opportunistic colonizers, including coagulase-negative staphylococci (n = 5, 5.1%), *Corynebacterium* spp. (n = 4, 4.0%), *Moraxella catarrhalis* (n = 4, 4.0%), and *Prevotella melaninogenica* (n = 4, 4.0%). Although generally considered part of the normal nasal microbiota, these organisms may acquire clinical relevance in immunocompromised hosts or in the context of polymicrobial biofilm-associated infections such as chronic rhinosinusitis.

*Escherichia coli* (n = 6, 6.1%) was classified separately as an enteric organism not typically resident in the sinonasal niche, and its presence may reflect secondary colonization or specimen contamination.

The data confirm that the sinonasal environment represents a complex reservoir of both commensal and opportunistic bacteria.

Analysis of resistance gene prevalence among the nasal and sinus isolates revealed that the most frequently detected determinants were *tem* (25.3%), *sul1* (21.2%), and *ermB* (20.2%). These were followed by *mecA* (10.1%), *shv* (9.1%), and *ctxM* (8.1%). Less common genes included *ndm* (5.1%) and *oxa48* (4.0%), while *oxa23*, *oxa24/40*, *kpc*, and *imp* were each identified in approximately 1% of the isolates.

Overall, β-lactamase-related genes (*tem*, *shv*, *ctxM*) represented the predominant class of resistance determinants, with macrolide (*ermB*) and sulfonamide (*sul1*) genes also frequently encountered. Carbapenemase-encoding genes (*ndm*, *oxa48*, *kpc*) were detected at low levels. The data indicate a wide distribution of multiple resistance mechanisms among sinonasal isolates, with β-lactam and macrolide resistance genes being the most prevalent in this cohort (Table 1).

Although carbapenemase genes (*oxa23*, *oxa48*, *ndm*, *imp*) were overall less prevalent, they occasionally co-occurred with ESBL-related genes (*ctxM*, *shv*, *tem*) in isolates of *Klebsiella pneumoniae* and *Proteus mirabilis*, suggesting sporadic introduction of multidrug-resistant Enterobacterales into the sinonasal niche.

**Figure 2:**
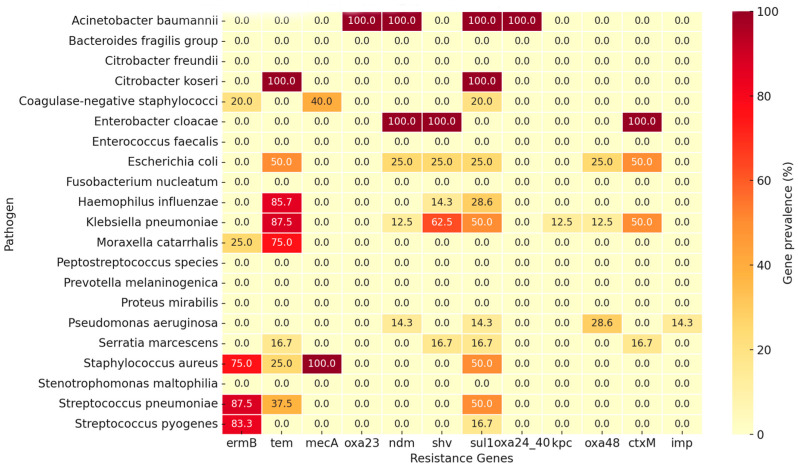
Distribution of resistance genes by pathogens. Gene prevalence is shown in percentages. Heatmap representation.

Analysis of antimicrobial resistance gene distribution among the nasal and sinus isolates revealed distinct species-specific patterns (Figure 2). Among Gram-positive isolates, *Staphylococcus aureus* displayed the highest overall gene prevalence, characterized by frequent detection of *mecA*, *ermB*, and *sul1*. *Streptococcus pneumoniae* primarily carried *ermB*, indicating a consistent macrolide-resistance profile. Within Gram-negative isolates, *Klebsiella pneumoniae*, *Proteus mirabilis*, and *Escherichia coli* exhibited elevated rates of β-lactamase-associated genes—particularly *tem*, *shv*, and *ctxM*—while carbapenemase determinants (*ndm*, *oxa48*) occurred sporadically. Overall, β-lactamase-related genes were the most widespread across pathogens, whereas macrolide and methicillin resistance genes predominated in Gram-positive species.

A co-occurrence analysis of the detected genes demonstrated that certain combinations appeared more frequently within the same isolate (Figure 2). The most common associations were *ermB*–*mecA*, *ermB*–*tem*, and *tem*–*shv*, representing concurrent macrolide and β-lactam resistance determinants. Co-detection of sul1 with β-lactamase genes (*tem*, *ctxM*) was also observed across several Gram-negative isolates. Carbapenemase genes, including *ndm*, *oxa48*, and *kpc*, showed limited overlap and occurred independently in a small proportion of samples. These patterns indicate the presence of multiple resistance determinants within individual isolates, suggesting a non-random distribution of genes across the sinonasal bacterial community.

Based on the distribution of resistance genes, distinct antimicrobial resistance profiles were observed among the major bacterial species isolated from nasal and sinus specimens. In Gram-positive isolates, *Staphylococcus aureus* exhibited gene patterns consistent with β-lactam and macrolide resistance, primarily through the detection of *mecA* and *ermB*, respectively. The presence of *mecA* indicates MRSA phenotypes, while *ermB* reflects resistance to macrolides, lincosamides, and streptogramin B antibiotics. *Streptococcus pneumoniae* similarly carried *ermB* as the dominant determinant, indicating a uniform macrolide-resistant profile within this group. In both species, co-occurrence of *ermB* and *sul1* suggests additional tolerance to sulfonamides in a subset of isolates.

Among Gram-negative organisms, resistance profiles were largely defined by β-lactamase production. *Klebsiella pneumoniae*, *Proteus mirabilis*, and *Escherichia coli* frequently harbored the *tem*, *shv*, and *ctxM* genes, corresponding to ESBL activity. These genes confer resistance to most penicillins and cephalosporins. The detection of *ndm*, *oxa48*, and *kpc* in a smaller subset of *Klebsiella* and *Pseudomonas aeruginosa* isolates indicates carbapenemase production, suggesting reduced susceptibility to carbapenems and last-line β-lactams. In contrast, *Haemophilus influenzae* showed limited gene diversity, mainly associated with *tem*, consistent with simple β-lactamase-mediated ampicillin resistance.

Overall, β-lactamase-related genes dominated among *Enterobacterales*, while macrolide and methicillin resistance markers were most frequent in Gram-positive cocci. Carbapenemase genes remained rare but were present in multiple genera, emphasizing the emergence of low-frequency but clinically relevant multidrug-resistant strains within sinonasal infections.

The analysis of gene co-occurrence among nasal and sinus isolates demonstrated that a limited number of resistance determinants tended to appear together within the same bacterial strains (Table 2). The most frequent associations involved *tem*–*sul1* (11.1%), *ermB*–*mecA* (7.1%), and *ermB*–*tem* (6.1%), indicating that β-lactamase- and macrolide-resistance genes were often present concurrently in individual isolates. Moderate co-occurrence was also observed between *tem*–*shv* (8.1%) and *ermB*–*sul1* (10.1%).

In contrast, carbapenemase-encoding genes (*ndm*, *oxa48*, *kpc*, *imp*) displayed minimal overlap with other gene families, generally appearing as isolated markers in single strains (<2%). Overall, the co-occurrence pattern indicated that multigene combinations were most prevalent among β-lactamase and macrolide resistance determinants, whereas carbapenemase genes remained sporadic within the sinonasal isolates.

The classification of nasal and sinus isolates according to multidrug resistance status revealed marked interspecies variability in antimicrobial susceptibility patterns (Table 3). Among Gram-positive organisms, *Staphylococcus aureus* displayed the broadest resistance profile, with a considerable proportion of isolates categorized as MDR or MRSA equivalents. These isolates were typically positive for the *mecA* gene and frequently co-carried *ermB* and *sul1*, indicating concurrent β-lactam, macrolide, and sulfonamide resistance mechanisms. In contrast, coagulase-negative staphylococci (CoNS) demonstrated mixed profiles, with the majority being susceptible or showing only SDR.

Among Gram-negative pathogens, *Klebsiella pneumoniae* exhibited a high proportion of MDR isolates, primarily due to the detection of ESBL genes such as *tem*, *shv*, and *ctxM*. *Proteus mirabilis* and *Escherichia coli* also demonstrated notable MDR frequencies, corresponding to ESBL-producing phenotypes, while *Citrobacter koseri* isolates were uniformly classified as MDR. A limited number of *Pseudomonas aeruginosa* and *Acinetobacter baumannii* strains were recovered; the former showed sporadic MDR or XDR profiles associated with carbapenemase genes (*ndm*, *oxa48*), while the latter was represented by a single XDR isolate.

Conversely, *Haemophilus influenzae* and anaerobic isolates such as *Bacteroides fragilis* group and *Citrobacter freundii* were predominantly susceptible, lacking multidrug resistance markers. These findings emphasize the heterogeneous resistance burden among sinonasal pathogens, with *Staphylococcus aureus* and *Enterobacterales* species representing the principal carriers of multidrug resistance, while other commensal or opportunistic flora maintained lower resistance profiles.

Review of patient data indicated that isolates classified as XDR or belonging to High-Risk MDR categories were predominantly recovered from patients with a history of repeated antimicrobial therapies (more than 5 over the course of 24 months) and maxillary sinus punctures. In contrast, isolates obtained from patients with less than 5 courses of antibiotic treatment in the past 24 months were more frequently categorized as low risk or moderate risk (SDR or susceptible strains).

In summary, the microbiological and molecular analysis of nasal isolates collected from CRS patients revealed a diverse bacterial spectrum with variable antimicrobial resistance profiles. β-lactamase-related genes (*tem*, *shv*, *ctxM*) and macrolide resistance determinants (*ermB*) were the most prevalent, while carbapenemase genes remained infrequent. Several bacterial species—particularly *Staphylococcus aureus* and *Klebsiella pneumoniae*—demonstrated multigene resistance patterns and were predominantly classified as MDR pathogens. The overall gene co-occurrence and MDR stratification analyses indicate that multiresistance within sinonasal microbiota is unevenly distributed across taxa, with a minority of high-risk isolates carrying multiple concurrent resistance determinants.

Correlation of resistance genes with phenotypic resistance

Among Gram-positive isolates, vancomycin and linezolid maintained universally low MICs (MIC50 0.75 and 1.0 µg/mL, respectively), confirming preserved activity against staphylococci and enterococci (Supplementary Table S1). In contrast, oxacillin showed uniformly high MICs (MIC50 = 4.0 µg/mL), consistent with a high proportion of *mecA*-positive MRSA isolates. Erythromycin MICs clustered at the resistance breakpoint (MIC50 = 8.0 µg/mL), reflecting widespread *ermB*/*ermC*-mediated macrolide resistance. Clindamycin demonstrated a bimodal distribution (MIC50 = 0.25, range 0.25–8 µg/mL), suggesting the presence of both inducible and constitutive MLSB resistance phenotypes.

Gram-negative isolates exhibited markedly higher MICs across β-lactam antibiotics (Supplementary Table S2). Third-generation cephalosporins (ceftazidime, ceftriaxone) and cefepime showed bimodal distributions with MIC90 values reaching 64 µg/mL, consistent with ESBL-producing Enterobacterales. Meropenem also demonstrated a bimodal pattern (MIC50 = 0.5, MIC90 = 64 µg/mL), reflecting a subpopulation of carbapenem-resistant isolates (likely *KPC* or *oxa-48* producers). Piperacillin and piperacillin–tazobactam showed high MIC50 values (32 and 128 µg/mL), suggesting limited utility for empiric therapy. TMP-SMX exhibited extreme variance (SD = 114), with MIC90 reaching 320 µg/mL, consistent with *sul1*-mediated sulfonamide resistance in a subset of isolates.

To assess whether the presence of specific antimicrobial resistance genes detected by Unyvero multiplex-PCR correlated with elevated MIC values measured by VITEK^®^2, Mann–Whitney U tests (one-tailed, gene-positive > gene-negative) were performed for each biologically plausible gene–antibiotic pair (Table 4). Only comparisons with ≥2 isolates in each group are reported.

Eight of 36 phenotype–genotype comparisons reached statistical significance (Table 4). The strongest correlations were observed for *mecA* with oxacillin resistance (MIC50 4.0 vs. 0.25 µg/mL, *p* < 0.001), *sul1* with TMP-SMX resistance (MIC50 320 vs. 10 µg/mL, *p* < 0.001), and *KPC* with meropenem resistance (MIC50 64 vs. 2.0 µg/mL, *p* < 0.001). The β-lactamase gene *tem* was significantly associated with elevated ampicillin and piperacillin MICs, confirming functional gene expression. The *ermC* gene correlated with clindamycin resistance but not erythromycin, suggesting that *ermC* in this cohort may predominantly mediate inducible MLSB resistance with greater impact on lincosamides. Notably, some expected correlations (e.g., *shv*/*ctxM* with cephalosporin MICs) did not reach significance, likely due to the confounding effect of intrinsic resistance in non-fermenting Gram-negatives or small subgroup sizes.

The concordance analysis (Table 5) revealed, as expected, that *mecA* demonstrated the highest agreement between genotype and phenotype (96.2%), with 100% specificity and 95% sensitivity for oxacillin resistance, supporting its use as a reliable molecular marker for MRSA screening. The *sul1*–*SXT* pair also showed high concordance (91.2%), although sensitivity was moderate (57.1%), indicating that not all *sul1*-positive isolates exceeded the high SXT breakpoint of ≥80 µg/mL. The low concordance for *ermB*–erythromycin (41.9%) reflects the complex regulation of *erm* genes, where gene carriage may not always translate to constitutive phenotypic resistance, particularly in isolates with inducible MLSB phenotypes.

**Figure 3:**
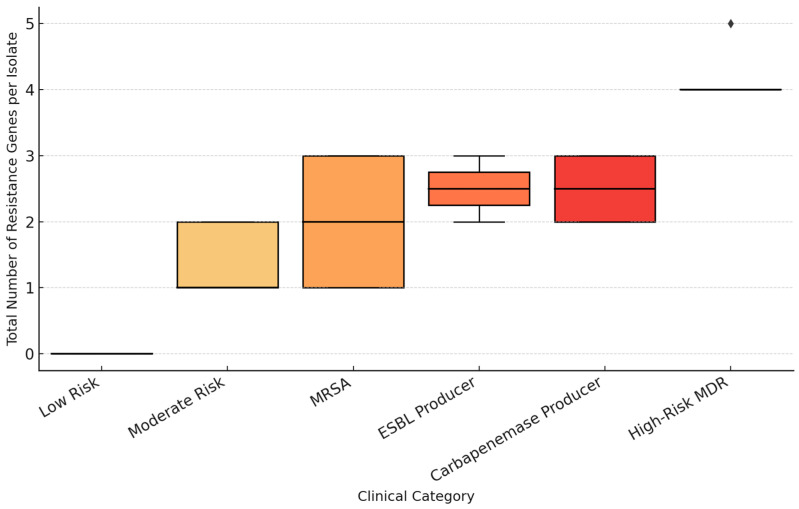
Total resistance gene burden by clinical category.

To explore the relationship between the genetic resistance load and the clinical risk level of nasal isolates, the total number of detected resistance genes per isolate was compared across predefined clinical categories (Low Risk, Moderate Risk, MRSA, ESBL Producer, Carbapenemase Producer, and High-Risk MDR). The resulting distribution demonstrated a clear, progressive increase in resistance gene burden with ascending clinical risk category (Figure 3). Isolates classified as Low or Moderate Risk carried few or no resistance genes, whereas MRSA and ESBL Producer categories displayed intermediate gene counts. The Carbapenemase Producer and High-Risk MDR groups exhibited the highest totals, indicating an accumulation of multiple resistance determinants in these isolates.

A significant progressive increase in the total number of detected resistance genes was observed across the clinical risk categories. Statistical analysis confirmed a strong positive correlation between the total resistance gene count and the phenotypic clinical risk level (Spearman’s rho = 0.966, *p* < 0.001). Isolates classified as High-Risk MDR or Carbapenemase Producers exhibited the highest accumulation of multiple determinants (e.g., co-occurrence of tem, shv, and ndm). Conversely, the Kruskal–Wallis test (H = 92.24, *p* < 0.001) demonstrated that variation in gene burden across categories was not due to chance, confirming that molecular load is a robust predictor of phenotypic resistance.

## 4. Discussion

Several limitations should be considered when interpreting our findings. The study population consisted exclusively of symptomatic patients with recurrent or treatment-refractory CRS, with a history of repeated antibiotic regimens or prior sinonasal interventions. As cultures were obtained only from this selected subgroup, the results may reflect a resistance-enriched cohort and may not be generalizable to the broader CRS population or to asymptomatic individuals. The absence of a healthy control group further limits the ability to determine whether the observed resistance gene prevalence represents disease-associated enrichment or background colonization within the sinonasal microbiota. In addition, sampling was cross-sectional and restricted to a single time point per patient, precluding evaluation of temporal resistance dynamics. Functional analyses, including assessment of gene expression, biofilm formation capacity, or transcriptional activity, were not performed, and whole-genome sequencing was not undertaken due to technological limitations; therefore, characterizations such as clonal relatedness and mobile genetic elements could not be assessed. Finally, predefined clinical risk categories incorporated phenotypic resistance profiles and selected molecular markers, introducing the possibility of partial circularity when correlating resistance gene burden with risk level. Although strong statistical associations were observed, these relationships should be interpreted within the given context, as risk stratification was not entirely independent of resistance parameters. Future multi-centre studies using independent clinical endpoints and longitudinal designs would be necessary to validate and expand upon our findings.

Bacteria play a significant role in the development and exacerbation of sinusitis symptoms, particularly in CRS. While the sinuses are typically sterile, bacterial colonization and infection can occur, contributing to the inflammation and persistence of sinusitis [15]. The presence of bacterial biofilms, particularly those formed by *Staphylococcus aureus* and *Pseudomonas aeruginosa*, is understood as a key factor in the chronic state of sinusitis, complicating treatment and exacerbating symptoms [14,15]. The role of bacteria in sinusitis is complex, involving interactions with host factors and other microbial populations, which can influence disease progression and treatment outcomes.

Healthy sinuses are generally sterile, but in cases of chronic sinusitis, bacteria such as *Staphylococcus aureus*, *Staphylococcus epidermidis*, and various *Streptococcus* species are commonly found in the sinuses and nasopharynx [34,35]. In CRS, bacteria are often present as colonizers rather than active pathogens, contributing to inflammation rather than direct infection [35]. The presence of bacterial biofilms, however, particularly those formed by *Staphylococcus aureus* and *Pseudomonas aeruginosa*, is associated with the persistence and exacerbation of CRS symptoms [36]. In some cases, Gram-negative or atypical bacteria may become culprits for persistent sinonasal disease [37,38].

Multiple studies account for bacterial implication in the pathogenesis of CRS, although the current understanding is that the condition is primarily characterized by chronic inflammation rather than infection [35,39]. The fine line between inflammation and infections is still being discussed and looked into. Anaerobic bacteria, such as *Prevotella*, *Fusobacterium*, and *Peptostreptococcus*, are often found in CRS patients, particularly when conditions favor their growth, such as reduced oxygen tension and increased acidity [40]. In addition to colonization, inflammation maintenance and increased acidity, the presence of bacterial superantigens, particularly from *Staphylococcus aureus*, may lead to TH2-mediated inflammation, exacerbating CRS symptoms [41]. Another factor is the presence of bacterial biofilms. Biofilms contribute significantly to the chronicity of sinusitis by protecting bacteria from the host immune response and antibiotic treatment, making infections more difficult to eradicate [36,42]. Biofilms are associated with more severe diseases, poorer surgical outcomes, and higher recurrence rates, making them a critical target for treatment strategies [43]. These multiple overlapping factors complicate the pathogenesis of CRS, making its primary driver difficult to pinpoint and study. Recent studies have provided deeper insights into the microbiological landscape of CRS, as bacteria are still believed to be the main culprit behind the disease. The most commonly identified bacterial pathogens in chronic sinusitis include *Staphylococcus aureus*, coagulase-negative staphylococci, and various anaerobic bacteria. *Staphylococcus aureus* is frequently isolated in CRS cases and is known for its ability to form biofilms, which contribute to persistent infections. MRSA is particularly prevalent, accounting for a significant portion of *Staphylococcus aureus* isolates in CRS [44,45]. CoNS are often considered contaminants, but these bacteria are the most common isolates in some studies and may play a pathogenic role in CRS as well [46]. Anaerobic Bacteria like *Prevotella*, *Fusobacterium*, and *Peptostreptococcus* species are commonly found in CRS, particularly in cases with a polymicrobial etiology [47]. *Streptococcus pneumoniae*, *Haemophilus influenzae*, and *Pseudomonas aeruginosa* are also frequently identified, with *Pseudomonas* being particularly common in nosocomial infections and immunocompromised patients [15,47]. Despite all these bacterial species, studies using 16S rRNA sequencing have shown that CRS is associated with a reduced bacterial diversity compared to healthy control subjects [48,49]. This reduced diversity may contribute to the disease’s chronic nature, in addition to certain bacterial culprits.

The discovery of specific antimicrobial resistance genes—particularly *tem*, *ermB*, *sul1* and *mecA* within the sinonasal microbiome of CRS patients—marks a significant shift in the clinical management of persistent disease. The results of this study demonstrate that the sinonasal cavity is not merely a site of inflammation but a reservoir for MDR and even XDR pathogens [29,50].

Currently, at a worldwide level, we are facing the crisis of conventional empiric therapy. The data reveal that β-lactamase-related genes (*tem*, *shv* and *ctxM*) and macrolide resistance markers (*ermB*) are the most prevalent in this cohort. This directly challenges current standard-of-care protocols defined in global guidelines [10,51]. In clinical practice, we most often see the failure of first-line β-lactams. The high prevalence of the *tem* gene (25.3%), especially when co-occurring with *ctxM* (8.1%) in *Klebsiella pneumoniae* and *Escherichia coli*, suggests that traditional empiric agents like amoxicillin–clavulanate are likely to fail. These genes indicate a transition toward ESBL phenotypes, which often require escalation to carbapenems or novel inhibitor combinations [43,47]. In addition to their antimicrobial role, macrolides are frequently utilized in CRS for their immunomodulatory properties. However, with detection of the *ermB* gene in 20.2% of isolates, the risk of selecting for even more resistant strains during therapy is high [52,53]. If a patient carries the marker, the antimicrobial benefit is neutralized, potentially leaving a bacterial reservoir that fuels type 2 inflammation [52,54].

A pivotal finding in this study is the correlation between the number of prior antibiotic courses and the resistance gene burden. Statistical analysis (Spearman’s rho = 0.966) confirms that isolates from patients with a history of repeated therapies harbor a substantially higher number of resistance genes. Clinicians should use “antibiotic history” as a proxy for molecular resistance. Patients with frequent past exposures should be fast-tracked to endoscopically guided middle meatal cultures or molecular PCR screening to identify markers typical for AMR before initiating further empiric cycles [55,56].

The co-occurrence of and in *Staphylococcus aureus* is particularly concerning within the CRS microbiome [57]. The capability of *Staphylococcus aureus* to form biofilms is well-known [18,20], and biofilms act as “hotspots” for horizontal gene transfer, where phenotypic tolerance and heritable resistance mechanisms coexist, rendering combined systemic therapy ineffective [58,59]. The proximity of bacteria within the extracellular polymeric substance of biofilms allows for the rapid spread of mobile genetic elements. When a patient is treated with a macrolide, the selection pressure favors the survival of *ermB*-positive strains. If these strains also carry *mecA*, the clinician inadvertently selects for a population that is resistant [60] to the two most common classes of antibiotics used in clinical otorhynolaryngology practice. The *mecA* gene, found in 10.1% of our isolates, was almost universally linked to an MDR status. *Staphylococcus aureus* remains a primary driver of CRS chronicity through its ability to form biofilms and secrete superantigens [29,61]. The presence of *mecA*-positive *Staphylococcus aureus* is often associated with higher SNOT-22 questionnaire scores and a higher rate of surgical sinus surgery revision [62]. For patients harboring *mecA* and *ermB*, systemic antibiotics often fail to reach the necessary inhibitory concentrations within a biofilm [63,64]. These findings may support a shift toward topical anti-microbial irrigations in clinical practice (e.g., mupirocin or gentamicin), which can deliver higher concentrations than systemic levels, potentially overcoming the genetic resistance mechanisms and resistant microorganisms [65,66].

Our phenotype–genotype analysis demonstrated that several resistance genes detected by Unyvero multiplex-PCR significantly correlated with elevated MIC values on VITEK^®^2. The strongest associations—*mecA* with oxacillin, *sul1* with TMP-SMX, and *KPC* with meropenem (all *p* < 0.001)—reflect well-established resistance mechanisms and support the biological validity of molecular detection as a rapid predictor of clinically relevant resistance. The *mecA*–oxacillin pair achieved the highest concordance (96.2%) with 95% sensitivity and 100% specificity, reinforcing its established role as a reliable molecular surrogate for MRSA screening. These findings are consistent with a large-scale blood culture study by Williams et al. (2020), which reported 93% genotype–phenotype concordance for *mecA* with oxacillin across 412 specimens [67]. Similarly, *sul1* showed high concordance with SXT resistance (91.2%) and perfect PPV, although moderate sensitivity (57.1%) suggests that the high CLSI breakpoint (≥80 µg/mL) and the polygenic nature of folate pathway resistance—involving additional *sul2* and *dfr* determinants not captured by the panel—may account for gene-positive isolates that remain phenotypically susceptible.

The failure of *shv* and *ctxM* to reach significance with cephalosporin MICs likely reflects limited statistical power in small subgroups combined with the confounding effect of intrinsic cephalosporin resistance in non-fermenting Gram-negatives, which elevated baseline MICs in the gene-negative comparator group. Likewise, the low concordance for *ermB*–erythromycin (41.9%) and the moderate concordance of *ermC*–clindamycin (80.6%) highlight the complexity of MLS_B resistance regulation. The stronger association of *ermC* with clindamycin rather than erythromycin suggests a predominance of inducible MLS_B phenotypes in our cohort, with important clinical implications for clindamycin therapy in staphylococcal infections where D-zone testing remains essential. The poor performance of *gyrA* for ciprofloxacin prediction (sensitivity 20%, PPV 35.7%) reflects the multifactorial nature of fluoroquinolone resistance, which typically requires cumulative mutations in both *gyrA* and *parC* topoisomerase genes alongside efflux-mediated mechanisms. Studies in both *Neisseria gonorrhoeae* and *Acinetobacter baumannii* have shown that combined *gyrA*/*parC* mutations are found in the vast majority of high-level fluoroquinolone-resistant isolates, while single *gyrA* mutations alone confer only modest MIC elevations [68,69].

Our findings demonstrate that rapid molecular detection provides clinically actionable information for select high-confidence gene–antibiotic pairs, particularly *mecA*–oxacillin and KPC–meropenem, where genotype reliably predicts high-level resistance. However, the variable concordance across different targets underscores that molecular and phenotypic approaches remain complementary rather than interchangeable. Previous evaluations of the Unyvero platform in pneumonia and bloodstream infection settings have similarly reported high specificity but variable sensitivity for resistance gene detection, with concordance rates ranging from 75% to over 90% depending on the gene target and specimen type [69]. Multiplex-PCR platforms can accelerate initial resistance profiling and guide early empiric therapy, but definitive antibiotic selection should continue to rely on confirmatory phenotypic susceptibility testing, especially for resistance mechanisms subject to complex regulation or multifactorial expression.

From a clinical point of view, these findings may provide a coherent explanation for the observed association between increasing antibiotic treatment courses and persistent sinonasal disease. The more antibiotic regimens fail, the higher the risk of a greater gene burden. Repeated antimicrobial exposure does not merely select single resistances in isolation but can enrich strains carrying linked determinants (*sul1* with β-lactamases; *mecA* with MLS resistance genes), thereby accelerating the emergence of multigene resistance profiles in the sinonasal microbiome [70,71,72]. The present findings support a shift in the clinical management of persistent CRS away from empirical antibiotic therapies and toward precision diagnostics coupled with antimicrobial stewardship. In a subset of patients—particularly those with repeated antibiotic exposure and prior sinonasal interventions—the sinonasal microbiome may become enriched with organisms carrying resistance determinants [73]. In this context, repeated empiric regimens are not only less likely to succeed but may further select for high-risk phenotypes [74]. The following key ideas summarize pragmatic, practice-oriented steps derived from our study, which we are currently studying and trying to implement within our current practice:

1. Stop “blind” repeats: If a standard course of amoxicillin–clavulanate fails, it is advisable not to rotate to a macrolide or a cephalosporin without a culture;
2. Identify the “high-risk” patient: Use the patient’s history of sinus punctures, sinus surgery and prior antibiotic use as a clinical suspicion marker for the presence of XDR or High-Risk MDR strains and for culture indication;
3. Consider preoperative molecular surveillance in these “high-risk” patients: Incorporate rapid PCR testing for *mecA* and carbapenemases in surgical patients to tailor post-operative antibiotic regimen.

## 5. Conclusions

This observational study characterizes the molecular resistance landscape of bacterial isolates obtained from patients with refractory CRS. β-lactamase-associated genes (*tem*, *shv*, *ctxM*) and macrolide resistance determinants (*ermB*) were the most prevalent resistance markers, whereas carbapenemase genes were infrequent. Significant genotype–phenotype concordance was demonstrated for selected resistance determinants, including *mecA*–oxacillin and *sul1*–trimethoprim–sulfamethoxazole, supporting the internal validity of the molecular findings.

Resistance gene burden increased progressively across predefined clinical risk categories and correlated strongly with multidrug resistance classification. Isolates categorized as MDR or XDR harbored significantly higher cumulative resistance gene counts compared to low- and moderate-risk strains. These findings indicate that refractory CRS may be associated with heterogeneous but quantifiable antimicrobial resistance profiles, particularly among *Staphylococcus aureus* and Enterobacterales species.

## Figures and Tables

**Table 1:** The most frequent resistance genes identified in the nasal isolates.

Gene	Count	Percent of Nasal Isolates	Associated Resistance Mechanism
*tem*	25	25.3%	β-lactamase (broad-spectrum penicillin resistance)
*sul1*	21	21.2%	Sulfonamide resistance (integron-associated)
*ermB*	20	20.2%	Macrolide-lincosamide resistance (ribosomal methylase)
*mecA*	10	10.1%	Methicillin resistance (MRSA marker)
*shv*	9	9.1%	Extended-spectrum β-lactamase (ESBL)
*ctxM*	8	8.1%	Extended-spectrum β-lactamase (ESBL)
*ndm*	5	5.1%	Carbapenemase (metallo-β-lactamase)
*oxa48*	4	4.0%	Carbapenemase (OXA-type β-lactamase)
*oxa23*/*oxa24_40*/*kpc*/*imp*	1 each	~1%	Carbapenemase/ESBL variants

**Table 2:** Co-occurrence of antimicrobial resistance genes in nasal isolates.

	*ermB*	*tem*	*mecA*	*oxa23*	*ndm*	*shv*	*sul1*	*oxa24_40*	*kpc*	*oxa48*	*ctxM*	*imp*
*ermB*	20.2%	6.1%	7.1%	0%	0%	0%	10.1%	0%	0%	0%	0%	0%
*tem*	6.1%	25.3%	2%	0%	1%	8.1%	11.1%	0%	1%	1%	5.1%	0%
*mecA*	7.1%	2%	10.1%	0%	0%	0%	5.1%	0%	0%	0%	0%	0%
*oxa23*	0%	0%	0%	1%	1%	0%	1%	1%	0%	0%	0%	0%
*ndm*	0%	1%	0%	1%	5.1%	2%	2%	1%	0%	2%	2%	0%
*shv*	0%	8.1%	0%	0%	2%	9.1%	5.1%	0%	1%	1%	6.1%	0%
*sul1*	10.1%	11.1%	5.1%	1%	2%	5.1%	21.2%	1%	0%	0%	4%	0%
*oxa24_40*	0%	0%	0%	1%	1%	0%	1%	1%	0%	0%	0%	0%
*kpc*	0%	1%	0%	0%	0%	1%	0%	0%	1%	1%	1%	0%
*oxa48*	0%	1%	0%	0%	2%	1%	0%	0%	1%	4%	2%	1%
*ctxM*	0%	5.1%	0%	0%	2%	6.1%	4%	0%	1%	2%	8.1%	0%
*imp*	0%	0%	0%	0%	0%	0%	0%	0%	0%	1%	0%	1%

**Table 3:** Antimicrobial resistance status by pathogen in nasal isolates.

Pathogen	MDR (%)	SDR (%)	Susceptible (%)	XDR (%)
*Acinetobacter baumannii*	0	0	0	100
*Bacteroides fragilis group*	0	0	100	0
*Citrobacter freundii*	0	0	100	0
*Citrobacter koseri*	100	0	0	0
*Coagulase-negative staphylococci*	20	20	60	0
*Enterobacter cloacae*	100	0	0	0
*Enterococcus faecalis*	0	0	100	0
*Escherichia coli*	25	25	25	25
*Fusobacterium nucleatum*	0	0	100	0
*Haemophilus influenzae*	28.6	71.4	0	0
*Klebsiella pneumoniae*	37.5	25	0	37.5
*Moraxella catarrhalis*	25	50	25	0
*Peptostreptococcus species*	0	0	100	0
*Prevotella melaninogenica*	0	0	100	0
*Proteus mirabilis*	0	0	100	0
*Pseudomonas aeruginosa*	28.6	14.3	57.1	0
*Serratia marcescens*	0	0	83.3	16.7
*Staphylococcus aureus*	50	25	0	25
*Stenotrophomonas maltophilia*	0	0	100	0
*Streptococcus pneumoniae*	62.5	25	12.5	0
*Streptococcus pyogenes*	16.7	66.7	16.7	0

**Table 4:** Statistically significant phenotype–genotype correlations.

Resistance Gene	Antibiotic	MIC50+	MIC50−	Mean+	Mean−	*p*-Value
*mecA* (MRSA)	Oxacillin	4.0	0.25	4.00	0.86	<0.001 ***
*sul1* (sulfonamide)	Sulfamethoxazole/thrimethoprim	320	10	320	24.5	<0.001 ***
*KPC* (carbapenemase)	Meropenem	64	2.0	53.3	5.66	<0.001 ***
*sul1* (sulfonamide)	Trimethoprim	16	0.5	16.0	2.71	<0.001 ***
*tem* (β-lactamase)	Piperacillin	128	32	128	58.1	0.001 **
*ermC* (MLSB)	Clindamycin	8.0	0.25	6.71	1.71	0.002 **
*tem* (β-lactamase)	Ampicillin	32	2.0	32.0	13.3	0.002 **
*gyrA* (FQ resistance)	Moxifloxacin	4.0	0.375	4.83	2.22	0.009 **

MIC50+ and MIC50− = median MIC for gene-positive and gene-negative isolates. Mann–Whitney U, one-tailed. *** *p* < 0.001, ** *p* < 0.01. Only significant results shown (8/36 comparisons).

**Table 5:** Concordance analysis: Molecular genotype vs. phenotypic resistance.

Gene → Phenotype	n	TP	TN	FP	Conc.(%)	Sens.(%)	Spec.(%)	PPV(%)	NPV(%)
*mecA* → OXA R	26	19	6	0	96.2	95.0	100	100	85.7
*sul1* → SXT R	68	8	54	0	91.2	57.1	100	100	90.0
*ermC* → CLI R	31	5	20	1	80.6	50.0	95.2	83.3	80.0
*KPC* → MEM R	38	9	20	0	76.3	50.0	100	100	69.0
*tem* → AMP R	24	8	10	0	75.0	57.1	100	100	62.5
*gyrA* → CIP R	77	5	43	9	62.3	20.0	82.7	35.7	68.3
*ermB* → ERY R	31	1	12	0	41.9	5.3	100	100	40.0

OXA = oxacillin; SXT = sulfamethoxazole/thrimethoprim; CLI = cyndamycin; MEM = meropenem; AMP = ampicillin; CIP = ciprofloxacin; ERY = erythromycin; TP = true positive (gene+ and phenotypically resistant); TN = true negative (gene− and susceptible); FP = false positive; FN = false negative. Concordance = (TP + TN)/total. PPV = positive predictive value; NPV = negative predictive value. Breakpoints as per CLSI 2025.

## Data Availability

The original contributions presented in this study are included in the article. Further inquiries can be directed to the corresponding author.

## Supplementary Materials

The following supporting information can be downloaded at: https://www.mdpi.com/article/10.3390/pathogens15030311/s1, Table S1: MIC distribution for Gram-positive isolates; Table S2: MIC distribution for Gram-negative isolates.

## Abbreviations

The following abbreviations are used in this manuscript:

CRS	Chronic rhinosinusitis
MRSA	Methicillin-resistant Staphylococcus aureus
QoL	Quality of life
AS	Acute sinusitis
CLSI	Clinical and Laboratory Standards Institute
SDR	Single-Drug Resistant
MDR	Multi-Drug Resistant
XDR	Extensively Drug Resistant
PDR	Pan-Drug Resistant
ESBL	Extended-Spectrum Beta-Lactamase
CoNS	Coagulase-negative staphylococci
AMR	Antimicrobial Resistance
