# Supplementary material for: Molecular Profiles and Antimicrobial Resistance Genes in Bacterial Isolates from Chronic Rhinosinusitis Patients
Source: Pathogens. 2026 Mar 12;15(3):311. doi: 10.3390/pathogens15030311 (PMC13029087; doi:10.3390/pathogens15030311)
Supplement: Supplementary file 1 [file pathogens-15-00311-s001.zip › pathogens-4157291-supplementary.pdf]

pathogens

MDPI

**Supplementary table S1.** MIC distribution for Gram-positive isolates.

| Antibiotic | Code | n | MIC50 | MIC90 | Range | Mean | SD | Interpretation |
|---|---|---|---|---|---|---|---|---|
| Ampicillin | AMP | 5 | 0.25 | 10.4 | 0.25–16 | 3.75 | 6.89 | Variable |
| Cefotaxime | CTX | 5 | 0.12 | 1.0 | 0.12–1 | 0.47 | 0.48 | Low MICs |
| Ceftriaxone | CRO | 5 | 0.12 | 2.8 | 0.12–4 | 1.07 | 1.68 | Low MICs |
| Gentamicin | GEN | 28 | 0.5 | 16 | 0–16 | 4.16 | 6.37 | Bimodal |
| Ciprofloxacin | CIP | 28 | 1.5 | 8 | 0.5–8 | 3.79 | 3.57 | Elevated |
| Levofloxacin | LEV | 5 | 0.5 | 0.8 | 0.5–1 | 0.60 | 0.22 | Susceptible |
| Moxifloxacin | MFX | 33 | 0.25 | 4 | 0–8 | 1.49 | 2.16 | Variable |
| Tetracycline | TET | 33 | 2.0 | 16 | 0.25–16 | 7.79 | 7.63 | High spread |
| Tigecycline | TGC | 33 | 0.12 | 0.45 | 0.06–1 | 0.20 | 0.23 | Susceptible |
| Chloramphenicol | CHL | 5 | 2.0 | 2.0 | 1–2 | 1.80 | 0.45 | Low MICs |
| Sulfamethoxa-zole/thrimetoprim | SXT | 28 | 10 | 40 | 10–320 | 27.1 | 59.3 | Outliers |
| Erythromycin | ERY | 33 | 8.0 | 8.0 | 0.12–8 | 5.14 | 3.67 | Mostly resistant |
| Rifampicin | RIF | 28 | 0.5 | 32 | 0.5–128 | 8.39 | 13.9 | Variable |
| Penicillin | PEN | 28 | 0.5 | 0.5 | 0.03–0.5 | 0.47 | 0.12 | Low MICs |
| Clindamycin | CLI | 33 | 0.25 | 8.0 | 0.25–8 | 2.53 | 3.47 | Bimodal |
| Linezolid | LNZ | 33 | 1.0 | 3.6 | 0–8 | 1.72 | 1.50 | Susceptible |
| Vancomycin | VAN | 32 | 0.75 | 1.0 | 0–2 | 0.83 | 0.47 | Susceptible |
| Teicoplanin | TEC | 28 | 0.5 | 4.0 | 0–32 | 3.25 | 8.18 | Outliers |
| Oxacillin | OXA | 28 | 4.0 | 4.0 | 0.25–4 | 3.08 | 1.62 | Mostly resistant |
| Fusidic acid | FUS | 26 | 0.5 | 32 | 0–32 | 8.27 | 11.6 | Variable |

MIC values in µg/mL. MIC50 = median inhibitory concentration; MIC90 = 90th percentile. S = susceptible, R = resistant.

**Supplementary table S2.** MIC distribution for Gram-negative isolates.

| Antibiotic | Code | n | MIC50 | MIC90 | Range | Mean | SD | Interpretation |
|---|---|---|---|---|---|---|---|---|
| Ampicillin | AMP | 24 | 32 | 32 | 2–32 | 19.5 | 15.1 | Mostly R |
| Amoxicillin/clavu-lanate | AMC | 27 | 32 | 128 | 2–128 | 41.3 | 48.5 | High spread |
| Ticarcillin | TIC | 32 | 128 | 128 | 8–128 | 98.3 | 49.3 | Mostly R |

| | | | | | | | | |
|---|---|---|---|---|---|---|---|---|
| Ticarcillin/clavula-nate | TCC | 33 | 64 | 128 | 4–128 | 75.6 | 54.6 | Mostly R |
| Cephalothin | CEP | 37 | 4.0 | 64 | 1–128 | 23.8 | 32.2 | Variable |
| Cefuroxime | CXM | 22 | 8.0 | 64 | 4–64 | 20.9 | 24.3 | Variable |
| Cefoxitin | FOX | 30 | 4.0 | 64 | 0.25–64 | 27.6 | 30.2 | Variable |
| Cefotaxime | CTX | 33 | 32 | 64 | 0–64 | 32.4 | 30.6 | High MICs |
| Ceftazidime | CAZ | 51 | 2.0 | 64 | 1–64 | 17.8 | 25.0 | Bimodal |
| Ceftriaxone | CRO | 53 | 2.0 | 64 | 1–64 | 21.3 | 28.6 | Bimodal |
| Cefepime | FEP | 55 | 2.0 | 64 | 1–64 | 21.9 | 29.6 | Bimodal |
| Aztreonam | ATM | 32 | 16 | 64 | 0.5–64 | 26.8 | 29.8 | High MICs |
| Ertapenem | ETP | 28 | 0.5 | 10.4 | 0.25–64 | 5.93 | 12.4 | Mostly S |
| Imipenem | IPM | 37 | 0.25 | 2.0 | 0.25–16 | 2.19 | 4.24 | Mostly S |
| Meropenem | MEM | 60 | 0.5 | 64 | 0.25–64 | 10.8 | 20.4 | Bimodal |
| Amikacin | AMK | 53 | 2.0 | 64 | 0–64 | 15.4 | 25.2 | Bimodal |
| Gentamicin | GEN | 72 | 1.0 | 16 | 0–64 | 4.98 | 9.36 | Variable |
| Tobramycin | TOB | 56 | 1.0 | 16 | 0–16 | 4.16 | 5.67 | Variable |
| Ciprofloxacin | CIP | 72 | 0.5 | 7.6 | 0–8 | 1.83 | 2.57 | Variable |
| Levofloxacin | LEV | 51 | 0.25 | 8.0 | 0.12–8 | 2.24 | 3.32 | Variable |
| Moxifloxacin | MFX | 45 | 0.25 | 8.0 | 0–8 | 2.11 | 2.92 | Variable |
| Tetracycline | TET | 45 | 1.0 | 16 | 0.5–16 | 5.49 | 6.29 | Variable |
| Tigecycline | TGC | 45 | 1.0 | 16 | 0.12–32 | 5.56 | 9.86 | High spread |
| Sulfamethoxa-zole/thrimetoprim | SXT | 57 | 10 | 320 | 0.5–320 | 61.7 | 114 | Extreme R |
| Colistin | COL | 30 | 0.5 | 16 | 0.5–16 | 3.65 | 6.29 | Bimodal |
| Piperacillin | PIP | 61 | 32 | 128 | 1–128 | 50.1 | 55.1 | High MICs |
| Piperacillin/tazo-bactam | TPZ | 31 | 128 | 128 | 4–128 | 78.8 | 59.0 | Mostly R |

MIC values in µg/mL. MIC50 = median inhibitory concentration; MIC90 = 90th percentile. S = susceptible, R = resistant.
